# Analysis of endophytic bacterial diversity in seeds of different genotypes of cotton and the suppression of Verticillium wilt pathogen infection by a synthetic microbial community

**DOI:** 10.1186/s12870-024-04910-2

**Published:** 2024-04-10

**Authors:** Chong-Die Wu, Yong-Bin Fan, Xue Chen, Jiang-Wei Cao, Jing-Yi Ye, Meng-Lei Feng, Xing-Xing Liu, Wen-Jing Sun, Rui-Na Liu, Ai-Ying Wang

**Affiliations:** 1 College of Life Sciences, Shihezi University, Shihezi, China; 2 Key Laboratory of Oasis Town and Mountain-Basin System Ecology, Xinjiang Production and Construction Corps, Shihezi, China

**Keywords:** High-throughput sequencing, Seed microbiome, Cotton Verticillium wilt, Microbial community construction, Synthetic Microbial Community (SynCom)

## Abstract

**Background:**

In agricultural production, fungal diseases significantly impact the yield and quality of cotton (*Gossypium spp.*) with Verticillium wilt posing a particularly severe threat.

**Results:**

This study is focused on investigating the effectiveness of endophytic microbial communities present in the seeds of disease-resistant cotton genotypes in the control of cotton Verticillium wilt. The technique of 16S ribosomal RNA (16S rRNA) amplicon sequencing identified a significant enrichment of the *Bacillus* genus in the resistant genotype Xinluzao 78, which differed from the endophytic bacterial community structure in the susceptible genotype Xinluzao 63. Specific enriched strains were isolated and screened from the seeds of Xinluzao 78 to further explore the biological functions of seed endophytes. A synthetic microbial community (SynCom) was constructed using the broken-rod model, and seeds of the susceptible genotype Xinluzao 63 in this community that had been soaked with the SynCom were found to significantly control the occurrence of Verticillium wilt and regulate the growth of cotton plants. Antibiotic screening techniques were used to preliminarily identify the colonization of strains in the community. These techniques revealed that the strains can colonize plant tissues and occupy ecological niches in cotton tissues through a priority effect, which prevents infection by pathogens.

**Conclusion:**

This study highlights the key role of seed endophytes in driving plant disease defense and provides a theoretical basis for the future application of SynComs in agriculture.

**Supplementary Information:**

The online version contains supplementary material available at 10.1186/s12870-024-04910-2.

## Background

The symbiotic relationship between plants and their microbiota holds profound significance in the fields of ecology and biology. Members of the microbiota promote plant growth and development and play a crucial role in the resistance to plant disease and environmental adaptation [1, 2]. Research has shown that bacteria in the plant seeds are competitive and easily colonize offspring plants [3, 4]. These bacteria may be vertically transmitted through seeds, which enables their stable propagation across generations [5–7]. From an evolutionary perspective, plants are likely to preferentially select bacteria with disease resistant or symbiotic traits for vertical transmission [8, 9]. Recent advances in research suggest that the beneficial microbes in some plant seeds can significantly optimize plant traits. Examples include beneficial microbes in the rhizosphere of drought-tolerant wheat (*Triticum aestivum*) and disease-resistant tomato (*Solanum lycopersicum*) genotypes [10, 11]. The study of ability of endophytic bacterial communities in cotton (*Gossypium spp.*) seeds to drive plant disease resistance merits further exploration. Therefore, a deeper exploration of the seed endophytic microbial communities and their functions will offer insights into unraveling their co-evolutionary mechanisms with plants and the development of functional microbial resources.

Seeds, as crucial carriers of species propagation and genetic information storage, have developed mechanisms to adapt to various types of stresses, which ensures the continuity of species [12–15]. Studies have shown that bacterial communities isolated from the seeds of multiple genotypes of rice (*Oryza sativa*) possess common traits related to adversity. This enables them to survive in the unique microenvironment of seeds [16]. Notably, although different studies have identified endophytic bacterial populations in the seeds of the same species, their abundance, diversity, and community structure may vary across studies. This can be owing to factors, such as seed structure, developmental stage, chemical composition, and genotype [17, 18]. This reflects the co-evolution of hosts and their seed microbiota [19]. Endophytic bacteria may not only affect the seed growth and development of cotton but also relate to agronomic traits, such as disease resistance and stress tolerance [20, 21]. Thus, in-depth research on the types, functions, and interactions of seed endophytic bacteria in cotton is of substantial theoretical and practical significance to improve resistance to disease in cotton and induce this trait.

As a globally important economic crop, cotton has broad applications in textiles, oil production, and medicine [22, 23]. Cotton Verticillium wilt, a devastating disease caused by the soilborne fungus *Verticillium dahliae*, severely affects the yield and quality of cotton [24]. *V. dahliae* was studied as the pathogen to determine the influence of cotton seed endophytes in plant disease resistance pathways. The prevalence of this disease in cotton has been exacerbated by continuous monoculture, and certain bacterial genera, including *Bacillus*, *Pseudomonas*, and *Enterobacter*, have been proven to contain species that can serve as biological agents against this disease [25, 26]. Endophytes isolated from the plant tissues enhance the plant's ability to resist invasion by pathogens [27, 28]. The utilization of these beneficial bacteria to construct SynComs is a promising strategy against soilborne pathogens and plant diseases, which can aid in promoting plant health [29]. Given this, this study explored cotton seed endophytes with a particular focus on their potential in combating *V. dahliae* by constructing synthetic seed endophytic microbial communities to address the defense against cotton Verticillium wilt in future cotton production.

In this study, the Verticillium wilt-resistant genotype Xinluzao 78 and the susceptible genotype Xinluzao 63 were selected as subjects. The diversity of seed endophytic bacterial communities in these two genotypes was analyzed, and specifically enriched strains isolated from the resistant genotype were evaluated for their functions with the goal of exploring the interactions between plants and seed endophytes. The objectives of this study were as follows: (1) to analyze the structure and function of seed endophytic bacterial communities in resistant and susceptible genotypes of cotton; and (2) to deploy an artificial synthetic community constructed from the seed microbiota of the resistant genotype in the seeds of the susceptible genotype to resist the occurrence of cotton Verticillium wilt.

## Materials and methods

### Experimental design and sample collection

The cotton seeds used in the study were collected from the experimental fields of Shihezi University in Xinjiang, China (44.30°N, 86.04°E), a region characterized by a temperate continental climate with an average annual temperature of approximately 8 °C and an average annual precipitation of 158.3 mm. Two different genotypes of cotton were used. They included Xinluzao 78 [30] with higher disease resistance and Xinluzao 63 [31] with minimal disease resistance. In May 2022, parent cotton plants were planted, and the offspring seeds were harvested in October 2022 to serve as experimental samples. Each cotton genotype was planted in four experimental plots. Each covered 18 m^2^ (3 m × 6 m) and was managed with 40 kg/ha of nitrogen (N), 25 kg/ha of phosphorus (P) in the form of P pentoxide (P_2_O_5_), and 20 kg/ha potassium (K) in the form of K oxide (K_2_O). A total of 25% of the N was used as a base fertilizer, with the remaining 75% applied in stages. The P and K fertilizers were applied as 70% and 50% base fertilizers, respectively, with the rest used as top dressing. Top dressing was applied eight times throughout the growing period following a 'one water one fertilizer' management strategy. After harvest, the seeds were delinted with sulfuric acid and stored at -4 °C. For each genotype, 10 seeds were mixed for an amplicon analysis. There were four biological replicates.

### DNA extraction and bacterial 16S rRNA gene amplification

Before the endophytic bacterial DNA was extracted from the seeds, the seed samples were first disinfected with 50 g/L sodium hypochlorite for 15 min followed by three rinses with sterile distilled water. The disinfected seeds were then soaked in sterile distilled water for 12 h and rinsed again three times. The final rinse water was used on agar plates to validate that the seeds had been thoroughly disinfected. The seed coat was removed using sterile tweezers, and the seeds were ground in liquid N under sterile conditions. The DNA was extracted using a DNeasy PowerSoil Kit (MP Biomedicals, Eschwege, Germany), and its purity and quality were assessed by 1% agarose gel electrophoresis. The samples were quantified using a NanoDrop One spectrophotometer (Thermo Fisher Scientific, Waltham, MA, USA). The DNA samples were stored at -80 °C.

The universal primers 335F (5’-CADACTCCTACGGGAGGC-3’) and 769R (5’-ATCCTGTTTGMTMCCCVCRC -3’) were used to amplify the V3–V4 region of the 16S rRNA gene to analyze the bacterial DNA diversity [20]. This PCR amplification utilized a Phanta Max Master Mix Kit P515 (Vazyme, Dalian, China) with optimized annealing temperatures, and the PCR products were verified using 1% agarose gel electrophoresis. The sample library was submitted to BMKCloud for quality inspection and sequenced on an Illumina NovaSeq 6000 platform (Illumina, San Diego, CA, USA) upon confirmation. The raw data were initially filtered using Trimmomatic (version 0.33) [32]. The primer sequences were identified and removed using Cutadapt (version 1.9.1) [33]. The paired-end reads obtained in the aforementioned steps were assembled using USEARCH (version 10) [34], and the chimeras were removed using UCHIME (version 8.1) [35]. High-quality reads that were generated from this process were used for subsequent analysis. Sequences ≥ 97% similar were clustered into the same amplicon sequence variants (ASVs) using USEARCH [32], and ASVs with a frequency < 0.005% were filtered out. The ASVs were taxonomically annotated using the SILVA database (version 132) [35] in the QIIME2 [36] naive Bayes classifier, with a confidence threshold of 70%. Sample rarefaction curves showed an asymptotic trend, which ensured that there was representative sample depth (Figure S1a). Eight seed samples were used to generate 1,280,404 raw reads in total with an average of 147,978 high-quality reads per sample. The RDP classifier [18] and SILVA database (version 138) [35] were used to successfully identify 1,135 bacterial ASVs from 396,164 reads, which included 19 bacterial phyla, 27 classes, and 538 species.

### Isolation, identification, and functional evaluation of the enriched strains specific to the resistant varieties

Specifically enriched endophytic bacteria were isolated from the resistant cotton seed genotypes starting with the surface disinfection of the seeds. The method for seed surface disinfection was the same as previously described. Ten de-coated seeds were ground in 5 mL of sterile distilled water. The suspension was treated in a 75 °C water bath for 15 min to eliminate non-Bacillus bacteria and then diluted tenfold before 10 µL was spread on LB agar plates. The plates were incubated at 30 °C for 2 d to grow the bacteria. Single colonies were picked onto new LB plates for purification. The purified isolates were transferred to LB liquid media that contained 15% glycerol and stored at -80 °C. The DNA was extracted from the strains for PCR amplification of the 16S rRNA gene using primers 27f (5'-AGAGTTTGATCMTGGCTCAG-3’) and 1492r (5'-GGYTACCTTGTTACGACTT-3') [37] followed by Sanger sequencing and a comparison using NCBI BLAST.

Plate confrontation methods were used to test the antagonism of isolated bacteria against *V. dahliae* V592. A volume of 50 µL of a spore suspension (1 × 10^7^ CFU/mL) was spread on potato dextrose agar (PDA), air-dried naturally for 10 min, and then symmetrically punched with a sterile borer (7 mm diameter). A volume of 10 µL of the endophytic bacterial suspension (1 × 10^7^ CFU/mL) was added to each well and incubated at 28 °C for 5 d. Sterile distilled water was added to the well of control group. These procedures on each strain were repeated three times. After 5 d, the antagonistic effect of each strain on V592 was observed. Compatibility testing of the strains utilized filter paper soaked in the liquid of each test strain. It was placed on plates spread with 50 μL of the target strains, incubated at 28 °C for 5 d and observed for any antagonistic inhibition.

### Selection and construction of an artificial synthetic community based on the broken-rod model and its suppressive effect on fusarium wilt

This study designed an experiment using the broken-rod model (Fig. 5A) to construct microbial communities of different types and abundances [38–41]. To analyze the relationship between strain functions and community diversity, a diversity gradient assembly was implemented, and each strain was randomly drawn. An alternative design strategy was used, which decreased the proportion of each single strain as the community richness increased. For example, the ratios for the 1-, 2-, 4- and 8-strain communities were 100%, 50%, 25%, and 12.5%, respectively.

A limited bacterial microsystem was established to study the suppressive effect of the endophytic bacteria in cotton seeds on Fusarium wilt. First, Xinluzao 63 cotton seeds were soaked in 70 ℃ warm water for 30 min and then for 8 h in 100 mL 1 × 10^7^ CFU/mL as described in Table S6. The seeds were then planted in cups filled with sterilized vermiculite and potting soil (1:3, v/v) with three seeds per cup. They were watered with a one-third strength MS nutrient solution and placed in a light incubator at 2,500 lx, with a 28 °C light/dark cycle of 16 h/8 h. Two days later, the wilt pathogen was inoculated into the microsystem, and then 50 mL of a V592 spore suspension (1 × 10^7^ CFU/mL) was added to the injured bottom roots through the bottom of the paper cup. The cotton was grown under conditions of 25 ℃ and 80% humidity. After 50 d, the disease index of the cotton plants was surveyed to preliminarily determine the composition of the microbial community. The selected microbial community combinations were subjected to functional validation by comparing seeds of the resistant cotton genotype Xinluzao 78 and susceptible genotype Xinluzao 63. The seeds of Xinluzao 63 seeds were soaked in the liquid of selected microbial community using the same method. Control groups were established with the seeds of both Xinluzao 78 and Xinluzao 63 that were soaked in sterile distilled water simultaneously. Three seeds were sown in each pot and thinned to one seedling after the cotyledon had unfolded. There were eight replicates. In parallel with investigating the suppression effect of the constructed microbial community on Fusarium wilt, the agronomic traits of the cotton plants were also examined, including measurements of the plant height, stem diameter, fresh root weight, and fresh stem weight, to assess the impact of the microbial community on cotton growth and development. The disease index was rated on a scale from 0 to 9 [42], where 0 indicated no symptoms, and 1, 3, 5, 7, 9 represented 0–10%, 11–25%, 26–50%, 51–75%, 76–90%, and > 90% discoloration or defoliation, respectively. The disease index was calculated as follows:

Disease index = [(grade × number of diseased plants) / total number of plants × highest grade] × 100; control effect = [(average disease index of the uninoculated group—average disease index of the inoculated group) / average disease index of the uninoculated group] × 100.

To isolate pathogens from the diseased cotton plants to fulfill Koch's postulates, the following steps were taken: 50 d after inoculating the pathogen, stems above the cotyledons of cotton plants were collected, surface-disinfected with 75% ethanol for 1 min, rinsed three times with sterile distilled water, treated with 10% hydrogen peroxide (H_2_O_2_) for 60 min, and rinsed three more times with sterile distilled water. The stems were cut into segments of approximately 1 cm and placed on PDA for 5 d at 28 °C.

### Verification of the colonization of the syncom using the antibiotic method

To test the colonization of the artificially synthesized microbial community in the cotton tissues, four bacteria from the selected community were activated. They were streaked onto solid media, and individual colonies were inoculated into 50 mL of LB liquid medium, followed by incubation at 28 °C in a constant temperature shaker at 180 rpm for 24 h. Four antibiotics that could inhibit the growth of the selected strains were identified by streaking the bacteria on plates with a concentration of 50 μg/mL. Subsequently, 1 mL of bacterial liquid was transferred to 50 mL of LB that contained 0.5 μg/mL of the antibiotic and cultured on a shaker. The culture was transferred every 24 h. The concentration of antibiotic was gradually increased as the liquid became turbid until it reached 50 μg/mL. Colonies that were consistent with those of the original strains were purified by selection on solid plates that contained this concentration of antibiotic and stored at 4 °C. The strains were passaged 10 times in antibiotic-free media 1 week later and then selected again on solid plates that contained 50 μg/mL of antibiotic to verify the stability of their antibiotic-resistant traits.

The cotton seeds were soaked for 8 h in the liquid of the marked antibiotic-tolerant artificially synthesized community (without antibiotics), and three seeds per pot were sown and grown until the two-leaf stage. There were three replicates. The plants were then tested to determine if they had been colonized by the strain. The entire cotton plant was dug out from the soil and cleaned, surface-disinfected by tissue type (root, stem, or leaf) using the same disinfection method that was used to isolate the pathogen, and then ground under sterile conditions. The bacterial liquid was diluted tenfold and inoculated onto LB solid plates that contained 50 μg/mL antibiotic and incubated at 30 °C for 3 d to determine whether the strain had colonized the plant.

### Statistical analysis and visualization

Statistical analyses were performed in R software (version 2023.06.1 + 524) [43], with all analyses using α = 0.05 as the level for significance. The false discovery rate (FDR) correction was applied for multiple hypothesis testing when required. Alpha-diversity analyses, adjusted for sparsity, were computed using the alpha function in the microbiome package (version 1.9.13) [44] in R to yield the ACE, Chao1, Shannon, and Simpson diversity indices. For the top 10 microbial communities with a relative abundance > 0.5%, a distribution visualization analysis was performed using the ggplot2 package (version 3.2.1) [45]. Inter-sample beta-diversity was assessed using the weighted UniFrac method, which considered the abundance and composition of ASVs, and visualized using a Principal Coordinates Analysis (PCoA) [46] in R to demonstrate similarities between the samples. Differences in the seed microbiomes between the two cotton genotypes were analyzed using MetagenomeSeq R (version v3.1.1; metagenomeSeq v1.22.0). This software assessed data on the abundance of species and normalized for annotation biases. It also accounted for the zero-inflation Gaussian distribution caused by sequencing depth. Based on this, significant differences were determined using linear models. The Tax4Fun2 tool (version v1.1.5) was used for functional gene prediction. The 16S sequencing data were classified at the species level based on the SILVA database (through QIIME or SILVAngs platform) and normalized for 16S copy numbers using NCBI genome data. A relationship between the SILVA classification and prokaryotic taxonomy in the Kyoto Encyclopedia of Genes and Genomes (KEGG) database was established to predict the KEGG functions of prokaryotic microbial communities. The SparCC algorithm was utilized to conduct an in-depth analysis of the bacterial community networks related to the cotton seeds. Networks were constructed at the class level and assessed the correlations between different classes to identify potential interactions in the microbial communities. Classes with an abundance > 0.01% and *P* < 0.05 were selected. Classes that appeared in at least 25% of the data were analyzed for network construction based on the similarity thresholds determined by the random matrix theory (RMT). A network analysis was conducted on the Molecular Ecological Network Analysis (MENA) platform [47], and the networks were visualized using Gephi software (version 0.9.2) [48].

## Results

### Analysis of the endophytic microbial community structure in cotton seeds

**Fig. 1:**
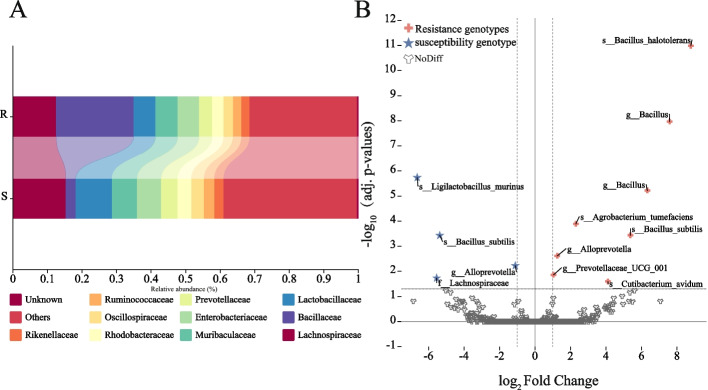
Based on the pyrophosphate sequencing of 16S rDNA amplicons, the structure of the endophytic bacterial community in cotton seeds was compared. **A** Bar chart of the microbiota composition, with species labeled at the family taxonomic level. **B** Volcano plot from the metagenomeseq analysis of endophytic bacteria in cotton seeds of two genotypes. Red indicates species enriched in resistant genotypes; blue represents species enriched in susceptible genotypes; gray signifies species with no significant difference in abundance. Points labeled in the plot correspond to significantly enriched species after adjustment. adj.*p*-value < 0.05 and |log2FC|> 1

Through 16S rDNA sequencing (Table S1), an average of 147,978 clean reads per sample was obtained. At 97% sequence similarity, the number of amplicon sequence variants (ASVs) ranged between 600 and 634. The species richness index showed no significant difference in the number of endophytic species between the Xinluzao 78 and Xinluzao 63 seeds. Despite similar species richness, the endophytic community in Xinluzao 63 seeds had a higher evenness, which indicated greater diversity (Figure S1B). There was a significant difference in beta-diversity between the two cotton seed genotypes with different degrees of resistance to disease. The PCoA demonstrated variations in the microbial communities between the seeds of different genotypes, with PC1 and PC2 explaining 59.9% and 23.39% of the total variation, respectively, for a total of 83.29% (Figure S1C). At the class level, Bacilli was the most abundant in the resistant genotype, while the abundance of Clostridia increased significantly in the susceptible genotype; it was less abundant in the resistant genotype (Figure S2A). At the family level, Bacillaceae was the most abundant in the resistant genotype Xinluzao 78 seeds (22.44%), while Lachnospiraceae was the most abundant in the susceptible genotype Xinluzao 63 (15.33%) (Fig. 1A). A linear discriminant analysis effect size (LEfSe) further confirmed that *Bacillus* is a key biological marker in the endophytic bacteria of resistant cotton seeds (Figure S1D). In the diversity analysis of the endophytic bacterial communities in the Xinluzao 78 and Xinluzao 63 seeds, 12 ASVs were identified with significant differences between the two genotypes (FDR-adjusted P ≤ 0.05) (Figure S3). The genera that were identified included *Bacillus*, *Agrobacterium*, *Alloprevotella*, *Prevotellaceae*, *Cutibacterium*, *Ligilactobacillus*, and *Lachnospiraceae*. Compared to the susceptible genotype Xinluzao 63, the proportions of *Bacillus* ASV4, ASV21447, ASV17937, and ASV2 (logFC, FDR-adjusted *P*-value: 8.78, 1.03 × 10–11; 7.59, 1.08 × 10–8; 6.33, 6.0 × 10–6; 5.37, 0.3 × 10–3), *Agrobacterium* ASV577 (2.30, 0.13 × 10–3), *Cutibacterium* ASV421 (4.11, 0.25 × 10–1), *Alloprevotella* ASV4461 (1.26, 0.24 × 10–2), and *Prevotellaceae* ASV12905 (1.05, 0.14 × 10–1) increased significantly in the resistant genotype Xinluzao 78, while *Ligilactobacillus* ASV20432 (− 6.64, 0.18 × 10–5), *Bacillus* ASV36823 (− 5.36, 0.37 × 10–3), *Lachnospiraceae* ASV29117 (− 5.54, 0.18 × 10–1), and *Alloprevotella* ASV685 (− 1.11, 0.60 × 10–2) decreased significantly (Fig. 1B, Table S2).

### Functional prediction analysis of the endophytic bacterial community in cotton seeds

**Fig. 2:**
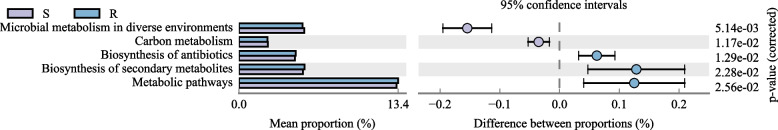
The illustrated figure delineates the differential analysis of KEGG metabolic pathways at the third level: Varied colors within the figure signify different groups. The left side displays the abundance ratio of distinct functions in either two individual samples or two sets of samples; the center exhibits the differential ratio of function abundance within the 95% confidence interval, while the value on the extreme right represents the *p*-value

To preliminarily understand the functional characteristics of the endophytic bacterial microbiome in cotton seeds, the Tax4Fun2 tool was utilized to normalize the classification results based on 16S rRNA, which referenced the genome annotations from NCBI. The establishment of a linear relationship between the SILVA classification and prokaryotic taxonomy in the KEGG database enabled prediction of the KEGG functions of the microbial community. In functional category level 3, a comparison of the top 10 functions in terms of abundance (Table S3) showed that the functional abundance of antibiotic biosynthesis averaged 4.77% in the Xinluzao 78 genotype; that of the biosynthesis of secondary metabolites was 5.51%, and the metabolic pathways accounted for 13.39%. Compared to the Xinluzao 63 genotype, these functions all showed significant differences in abundance (*P* ≤ 0.05) (Fig. 2). In the Xinluzao 63 genotype, the functional abundance of metabolism in the various environments averaged 5.50%, and that of carbon metabolism was 2.43%. The differences compared to Xinluzao 78 were significant at 0.15% and 0.03%, with a 95% confidence interval of 0.114 to 0.195, and the *P*-value adjusted to 0.0051 (*P* ≤ 0.05) (Fig. 2).

### Correlation network characteristics of the endophytic bacteria in resistant and susceptible cotton seeds

**Fig. 3:**
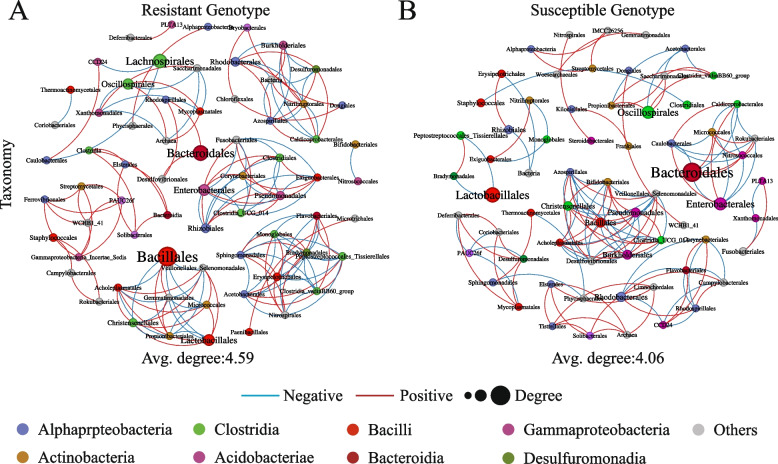
Network visualization of the interaction architecture within endophytic bacterial communities of resistant and susceptible genotypes of cotton seeds. **A** Resistant genotype Xinluzao 78. **B** Susceptible genotype Xinluzao 63

Two bacterial correlation networks were constructed to explore the bacterial interactions within the cotton seeds of different levels of resistance (Fig. 3). Overall, these networks predominantly exhibited positive correlations among the bacterial taxa. A deeper analysis of the network topological parameters revealed that the network complexity of Xinluzao 78 (mean degree index of 4.59) was higher than that of Xinluzao 63 (mean degree index of 4.06). Additionally, the network of Xinluzao 78 showed higher levels of modularity and a greater ratio of positive to negative correlations, which indicated a more complex microbial network structure with tighter interconnectivity. In the endophytic bacterial network of the disease-resistant genotype Xinluzao 78, the order Bacillales emerged as a key node with the highest connectivity. In contrast, the susceptible genotype Xinluzao 63 featured the order Bacteroidales as its key node (Table S4).

### Identification of endophytic bacteria in the cotton seeds and assessment of their plant growth-promoting characteristics

**Fig. 4:**
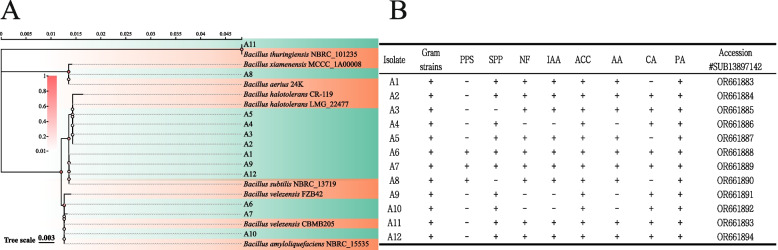
Phylogenetic classification and PGP characterization of 12 representative isolates cotton endophytic bacteria. **A** Phylogenetic tree was constructed based on the 16 S rDNA sequences of endophytic bacteria from cotton seeds and their close model strains. MEGA11 software (version 11.0.10. https://www.megasoftware.net/.) was used. Evolutionary distances were calculated using the Kimura 2-parameter method, and trees were constructed using the minimum evolution method. **B** PGP trait results for 12 representative isolates. Positive and negative results for each trait are indicated by " + " and "-," respectively.Gram strains, Gram stain; PPS, phosphate solubilization; SPP, iron carrier production; NF, nitrogen fixation; IAA, indoleacetic acid production; ACC, 1-aminocyclopropane-1-carboxylic acid deaminase production; AA, amylase; CA, cellulase; PA, pectinase

**Fig. 5:**
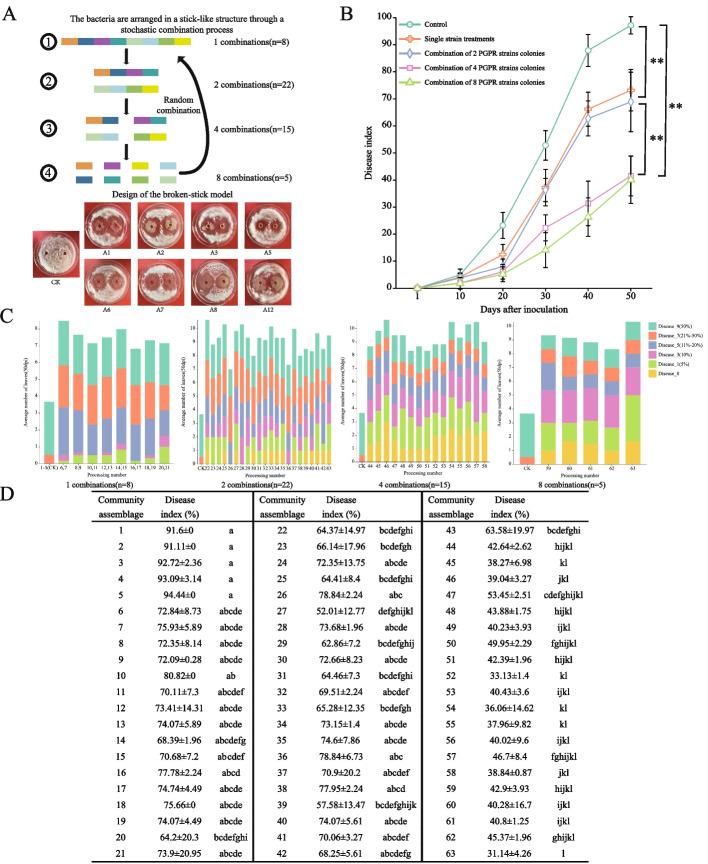
Construction of synthetic microbial communities guided by the broken-stick model and their disease progression trend in a limited microbial environment. **A** Schematic representation of the broken-stick model and the inhibitory effect of seed endophytes on the expansion of pathogenic fungal mycelium 5 days post-inoculation. **B** In sterilized nursery soil, the Xinlu Early 63 cotton was treated with the synthetic microbial community, showing its progression against fungal wilt disease. Repeated measures ANOVA revealed a significant difference between the cotton treated with microbial strains and the untreated control group (*P* < 0.001). Additionally, there was a significant difference in the number of microbial strain combinations between 1, 2 and 4, 8 (*P* < 0.001). **C** On the 50th day post-infection, the severity of the disease in the cotton plants is displayed. The treated cotton underwent three repeated measurements to calculate the disease index and severity. The data shown represents the average of three biological replicates along with their standard deviation. (ANOVA, ** indicates *P* < 0.001). **D** Biocontrol of cotton fungal wilt by different PGPR community combinations.Different lowercase letters within the same column indicate significant difference at the 0.05 level

The differences in community abundance between the endophytic bacterial communities in the resistant and susceptible genotypes of cotton seeds were compared using MetagenomeSeq. This resulted in the identification of 12 ASVs with significant differences. In the resistant genotype, the significantly enriched ASVs predominantly belonged to *Bacillus*, with a high proportion in both number and abundance. Thus, the strains of this genus were identified as the focus of this study. Strains that were specifically enriched were isolated from the seeds of the resistant genotype, and their 16S rRNA sequences > 99% similarity with the reference strains. A total of 12 strains of Bacillus strains were successfully isolated and purified (Fig. 4A). The strains were identified further and screened for their antagonistic ability against *V. dahliae* V592. Eight strains were significantly antagonistic with compatibility tests that did not identify any antagonism among the strains (Figure S4), which suggested that they could be developed as potential biocontrol agents (Fig. 5A, Table S5). Physiological and biochemical tests of these eight strains revealed that they could all produce ACC deaminase; 75% grew in an environment free of N; 25% had the ability to dissolve insoluble phosphates, and 75% could produce siderophores. Most of the strains could produce indole-3-acetic acid (IAA) and secrete amylase and cellulase, and all the strains exhibited pectinase activity (Fig. 4B). These eight strains can be considered to be Plant Growth-Promoting Rhizobacteria (PGPR) strains.

### Construction of a syncom using the broken-rod model and validation of its effectiveness in controlling verticillium wilt

In the limited-bacteria microsystem, 16 independent PGPR strain treatments (i.e., microbial combinations 6 to 21) achieved an average disease index of 73.19 (Fig. 5D). Among these, treatments A5 and A8 (i.e., microbial combinations 14, 15, 20, and 21) had relatively lower disease indices, at 69.54 and 69.05, respectively, while treatment A2 (i.e., microbial combinations 16 and 17) had a disease index of 76.26. In the microbial combinations that were composed of two types of PGPR (i.e., microbial combinations 22 to 43), the average disease index was 68.89. Notably, microbial combination 27 had the lowest disease index, and it was significantly lower than that of the other microbial treatments, such as combinations 26 and 36. The microbial combinations that consisted of four PGPR strains (i.e., microbial combinations 44 to 58) had an average disease index of 41.13, with no significant difference in disease indices among these combinations. They were significantly lower than most of the treatments that consisted of one or two strains. This was similar to the disease indices of the microbial combinations that were composed of eight PGPR strains (Fig. 5B and C). Considering the cost of production and controllability of the bio-formulations, one of the four-strain combinations was selected for subsequent experimental validation. Microbial combination 46 was chosen for further validation experiments when the disease index, leaf disease distribution, and cost of production were considered.

### Functional validation of SynCom and their colonization status within the host

**Fig. 6:**
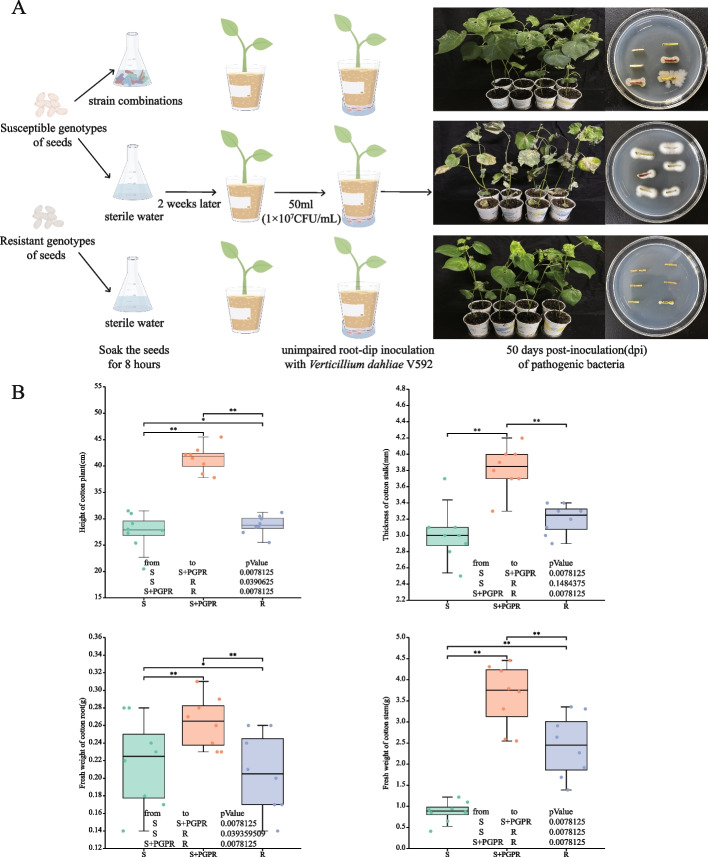
Disease resistance trials with synthetic communities and agronomic trait studies. **A** Experimental program to functionally validate synthetic microbiota infiltrating susceptible cotton genotypes. **B** Biostimulatory effects of synthetic communities on agronomic traits in susceptible cotton genotypes. Plant height (cm), stem thickness (mm), root fresh weight (g), and stem fresh weight (g) (ANOVA, * indicates *P* < 0.05; ** indicates *P* < 0.001). Asterisks indicate significantly longer inoculation-promoting effects. Boxes indicate interquartile ranges (75% to 25% of data, *n* = 8), and median values are shown as a line in the box

To delve deeper into the functional characteristics of the artificially synthesized communities, a seed soaking inoculation experiment on the susceptible genotype Xinluzao 63 cotton was conducted and compared with two different resistance genotypes of cotton to verify the function of the synthetic community. The results showed that the disease index of Xinluzao 63 inoculated with the synthetic community averaged 28.15%. The disease indices of uninoculated Xinluzao 63 and Xinluzao 78 averaged 84.69% and 29.14%, respectively. The average effectiveness of control in Xinluzao 63 inoculated with the synthetic community was 66.17%. Pathogen recovery tests indicated that *V. dahliae* was not isolated from the stems of cotton inoculated with the synthetic community, while fungal mycelia grew in the stems of uninoculated Xinluzao 63 (Fig. 6A). Agronomic trait surveys further confirmed that compared to the more resistant Xinluzao 78, the synthetic community significantly increased the plant height by 43.58%, stem thickness by 19.53%, fresh weight of the stem by 48.49%, and fresh weight of the root by 27.88% (all *P* < 0.001) of Xinluzao 63 (Fig. 6B).

The migration and colonization of PGPR strains in the plant were studied after the seeds had been inoculated by soaking. Four PGPR strains with different antibiotic resistance were successfully isolated from the inoculated cotton plants. These strains colonized different tissues of the cotton plant to varying extents, with A1 and A3 primarily colonizing the cotton leaves, and A8 and A12 colonizing within the cotton plant (Figure S4).

## Discussion

This study utilized high-throughput sequencing technology to thoroughly analyze the diversity of endophytic microbial communities in disease-resistant and susceptible genotypes of cotton seeds. A functional prediction analysis in the seeds of disease-resistant cotton genotypes revealed a rich array of microbial functions related to the biosynthesis of antibiotics and other secondary metabolites and other key metabolic pathways. An inter-group difference analysis was combined with traditional strain culture and isolation techniques and the broken-rod model to successfully construct a SynCom based on specifically enriched strains in the disease-resistant genotype. The results show that this synthetic community not only significantly enhanced the disease resistance of the susceptible cotton genotype but also effectively colonized the tissue parts of the cotton, which promoted its growth and development. Based on these findings, a new strategy of using endophytic microbial communities with specific phenotypes from plant seeds to develop bioformulations that drive certain resistance in plants was proposed.

### The microbiome of resistant and susceptible genotype cotton seeds

The microbial communities of seeds can be vertically transmitted from parent plants to offspring and dynamically regulate germination and plant growth. Recent studies have confirmed that bioformulations based on the seed microbiomes can effectively enhance plant growth and health [49, 50]. In this study, the endophytic microbial communities of disease-resistant and susceptible genotype cotton seeds were analyzed. Significant differences in the seed endophytic bacterial microbiomes between these genotypes were observed (Figure S1). Previous research has shown that even within the same species, the abundance, diversity, and structure of the dominant endophytic communities can vary between studies [18, 51]. This could be related to factors, such as seed structure, parental growth environment, and host genotype [52, 53]. Furthermore, studies suggest that the seed microbiome composition has species-specific and domestication traits and is associated with its protective role against plant pathogens [54]. This study further revealed that different cotton genotypes affect the composition of bacterial microbiomes (Figure S1C), which indicated the necessity of in-depth studies on the microbial communities within the seeds of cotton genotypes with quality agronomic traits.

The analysis of microbiome diversity indicated that although there was no difference in the species richness index of endophytic bacteria between the two genotypes of cotton seeds, the endophytic community in the susceptible genotype Xinluzao 63 was more diverse. This finding is consistent with those of previous studies on cucumber (*Cucumis sativus L.*) [55] and cotton [56]. We hypothesized that the higher microbial bacterial diversity in the susceptible genotype seeds could stem from the complexity of their internal ecological environment. Compared to the resistant genotype, the susceptible genotype is more influenced by external microbial environments, which leads to enhanced diversity in its microbial community structure [57]. Additionally, the abundant microbial metabolism and carbon metabolic activities in the susceptible genotype (Figure S3b) also corroborate its higher tolerance to various microbial populations, which could be a significant reason for its high microbial diversity. The susceptible genotype could lack strong selectivity to repel certain microbes, which enabled the coexistence of a broader range of microorganisms [58]. In addition, compared to the susceptible genotype, the microbial network of the resistant genotype had a higher degree of network modularity and a ratio of positive to negative correlations, which is consistent with the findings of previous research [59–61]. Notably, highly interconnected and modular microbial communities provide a potential basis for the plant immune system to rapidly activate defense responses against pathogens [62].

### Association of specific bacteria in the resistant cotton genotypes with resistance phenotypes

Understanding the composition of endophytic bacterial communities in disease-resistant and susceptible genotype cotton seeds is crucial to effectively utilize seed microbiomes to enhance plant growth and defense capabilities. Previous studies have revealed that cotton seeds can rapidly establish endophytic bacterial communities after germination whether these bacteria originate from the seeds themselves or from external soil [63–65]. Genetic factors and morphological/physiological effects may cause significant differences in the bacterial endophytic communities among different genotypes during the growth and development of cotton seedlings. This study showed that Lachnospiraceae, Lactobacillaceae, Muribaculaceae, and Enterobacteriaceae are the primary families in the cotton endophytic bacterial community with abundances > 5% (Figure 1A). Additionally, there were differences in the endophytic bacterial communities of the resistant and susceptible genotype cotton seeds. The seed microbiomes originate from diverse sources, including the soil, stems, leaves, flowers, and fruits, and these microbes are gradually selected and filtered in different plant compartments. Endophytes can enter the seeds through various pathways [66, 67]. Plants can also recruit specific microbiomes and attract beneficial microbes for colonization by releasing root exudates, such as amino acids, nucleotides, and long-chain organic acids. The results of functional prediction analysis showed that endophytic bacteria in the seeds of resistant genotype significantly differed from those of the susceptible genotypes in metabolic pathways related to the biosynthesis of antibiotics and secondary metabolites, which potentially enhanced the plant's adaptability and survival (Figure S3B).

Microbial communities in the plant seeds exhibit unique adaptability and colonization abilities owing to their special ecological niches [15, 68]. The traits of beneficial microbes in the seeds reflect the selection preferences of the host plant for endophytes [16]. In this study, an analysis of the differences in seed microbiomes between the resistant and susceptible genotypes indicated that seeds of the resistant genotype significantly enriched *Bacillus*, while the susceptible genotypes primarily enriched *Ligilactobacillus* (Fig. 1B). These enriched microbes may play a core role in inducing the plants to resist diseases [69]. Further experiments validated this since the eight strains of *Bacillus* isolated from the resistant genotype cotton showed a significant degree of pathogen inhibition and beneficial physiological and biochemical properties for plant growth and development (Fig. 3A, B). Recent studies have reported the enrichment of potentially beneficial endophytic bacteria, such as *Paenibacillus* and *Bacillus* [70, 71], in some plant seeds. These microbes may preferentially colonize seeds, thus, providing a barrier against pathogens. This priority effect where early colonizing microbes can limit or prevent the colonization of pathogens, thereby offers protection against pathogens [72]. Overall, microbial communities within seeds play a vital role in the plant lifecycle, not only aiding in plant growth and development but also playing an irreplaceable role in resisting pathogens. It will be possible to more effectively utilize these microbial communities to promote plant growth and health by gaining a deeper understanding of the composition and functions of these microbial communities.

### Control strategies for plant diseases using artificial synthetic communities constructed with the broken-rod model

Species of *Bacillus* have numerous advantages in biological control since they grow rapidly, are highly adaptable, and can produce a variety of beneficial antimicrobial compounds [73, 74]. They have been extensively studied and used in biological control. In plant-microbe interactions, rich bacterial diversity is often associated with enhanced resistance to pathogen invasion, which is primarily achieved through competition for resources [75]. Additionally, the diversity and composition of the ecological communities also affect the successful invasion of other organisms [76]. In particular, rich species diversity indicates that there are more ecological niches in the community, which not only enhance the community's adaptability in the unstable rhizosphere environment, but also help enable the bacteria to colonize the rhizosphere over the long-term [77]. More importantly, rich ecological niches enhance the competition between the bacterial communities and potential pathogens, thereby providing better protection for the host plant [78]. Considering the growing need for the production of cotton on limited land, more efficient plant disease control methods are required. Microbial inoculants, such as disease-resistant bacteria and pathogen competitors, are receiving widespread attention as future alternatives to chemical pesticides. Interestingly, the abundance of bacteria plays different roles in enhancing plant defense capabilities [79], which significantly increases the difficulty of selecting effective beneficial microbial communities. Although species-rich communities can more efficiently utilize resources and increase ecological efficiency, intense internal competition may negatively impact these benefits [80]. For this reason, this study proposes an ecological framework based on the broken-rod model with the goal of assembling effective probiotic community functions.

The broken-rod model was used to successfully screen a microbial community that was composed of four strains of Bacillus (A1, A3, A8, and A12) that were selected from eight antagonistic strains. Under indoor cultivation conditions, this community significantly enhanced the resistance of susceptible cotton genotypes to Verticillium wilt (Fig. 5A). Notably, after treatment with this synthetic community, several agronomic traits of cotton, including plant height, stem thickness, fresh weight of the stem, and fresh weight of the root, were significantly improved. This finding is consistent with recent trends in research. For example, Niu et al. (2017) [81] found that the application of combined microbial strains often surpasses the performance of a single strain in combating plant diseases. Similarly, Carrión et al. (2019) [82] revealed that the numbers of beneficial microbes rapidly increase when the plants are threatened by pathogens, which effectively constructs a synthetic ecology to suppress diseases. An antibiotic marking method was used to further explore the ability of the artificially constructed community to colonize cotton. The results showed that these four bacterial strains colonized different parts of the cotton plant, such as the leaves, stems, and roots (Figure S4). Overall, this study confirms that this artificial synthetic community not only successfully colonized susceptible cotton genotypes but also achieved significant effects in enhancing resistance to Verticillium wilt. In the future, we plan to investigate how this community promotes the disease resistance of cotton at the molecular level and explore its broad value for application in agriculture.

## Conclusion

This study analyzed the diversity of endophytic microbial communities in cotton seeds of different levels of resistance using high-throughput sequencing technology, which revealed their key role in driving disease resistance in cotton. Beneficial microbes, such as Bacillus, in resistant cotton seeds play a crucial role in defending against Verticillium wilt. The study indicates that specific Bacillus strains isolated from cotton seeds can significantly antagonize *Verticillium dahliae*. An artificial microbial community that significantly enhances resistance to cotton Verticillium wilt was constructed through mathematical modeling. It improved the growth condition of the cotton plants, and enabled seed endophytes to occupy ecological niches and colonize plant tissues, thus, laying the foundation for the future development and utilization of composite microbial formulations. These results highlight the substantial potential of using seed endophytic microbes for disease resistance in sustainable agriculture. Since most experiments were conducted in greenhouses, validation under actual field conditions is an important direction for future research.

### Supplementary Information

**Additional file 1: Supplementary Fig. 1.** Structure of seed endophytic microbiota based on 16S rDNA amplicon pyrosequencing. (A) Comparison of sample rarefaction curves. Species richness was calculated at the 3% dissimilarity level.(B) Comparison of diversity indices of samples at the 3% dissimilarity level. ACE and Chao represent richness diversity indices. Simpson and Shannon represent evenness diversity indices.(C) Principal Coordinate Analysis. Principal Coordinate Analysis (PCoA) was performed at the 3% dissimilarity level using representative sequences. The PCoA plot shows clear differences between the microbiotas of susceptible variety Xinlu Zao63 and resistant variety Xinlu Zao78.(D) In the comparison of endophytic bacterial communities between resistant and susceptible cotton seeds using LEfSe (Linear Discriminant Analysis Effect Size), only the top four most distinct biomarker taxa are displayed.

** Additional file 2: Supplementary Fig. 2.** Taxonomic units sorted by different varieties, where R represents the resistant variety Xinluzao78, and S represents the susceptible variety Xinluzao63: (A) Class; (B) Order; (C) Family; (D) Genus.

** Additional file 3 Supplementary Fig. 3.** Analysis of Between-group Differences in Community Variations. The MetagenomeSeq method was employed to scrutinize disparities in microbial community abundance between two cotton varieties, conducting taxonomic comparisons at the OTU (Operational Taxonomic Unit) level. Normalization was utilized to mitigate biases in taxonomic annotation, and the zero-inflated Gaussian distribution was applied to counteract the effects of sequencing depth. Subsequent analyses utilized linear models to identify significant differences. Statistical assessments were conducted using one-way analysis of variance (ANOVA) and Kruskal–Wallis tests within SPSS software. Notably, 12 bacterial categories demonstrated significant p-values (**, p < 0.005).

** Additional file 4: Supplementary Fig. 4.** Bacterial Strain Affinity Identification.

** Additional file 5: Supplementary Fig. 5.** Antibiotic method for detecting strain colonization. In the recovery experiment on different parts of cotton, four PGPR strains selected by antibiotics were presented. The first three images showed the smear photos of various parts of cotton treated with PGPR strains that were not selected by antibiotics.

** Additional file 6.**

** Additional file 7.**

** Additional file 8.**

** Additional file 9.**

** Additional file 10.**

** Additional file 11.**

## Data Availability

The dataset provided in this study can be found in an online repository. The name of the repository and the accession number can be found below: NCBI (Accession Number: PRJNA1031440).

## Abbreviations

16S rRNA: 16S ribosomal RNA
SynCom: Synthetic Microbial Community
*V. Dahliae*: *Verticillium dahliae*
PCR: Polymerase Chain Reaction
LB: Luria-Bertani
PDA: Potato Dextrose Agar
CFU/mL: Colony-Forming Units per milliliter
MS: Murashige and Skoog medium
FDR: False Discovery Rate
ACE: Abundance-based Coverage Estimator
PCoA: Principal Coordinates Analysis
SparCC: Sparse Correlations for Compositional data
RMT: Random Matrix Theory
MENA: Molecular Ecological Network Analysis
ASVs: Amplicon Sequence Variants
LEfSe: Linear Discriminant Analysis Effect Size
logFC: Log Fold Change
ACC: 1-Aminocyclopropane-1-Carboxylic Acid
IAA: Indole-3-Acetic Acid
PGPR: Plant Growth-Promoting Rhizobacteria
