# Supplementary figures and images for: Analysis of endophytic bacterial diversity in seeds of different genotypes of cotton and the suppression of Verticillium wilt pathogen infection by a synthetic microbial community
Source: BMC Plant Biol. 2024 Apr 10;24:263. doi: 10.1186/s12870-024-04910-2 (PMC11005247; doi:10.1186/s12870-024-04910-2)

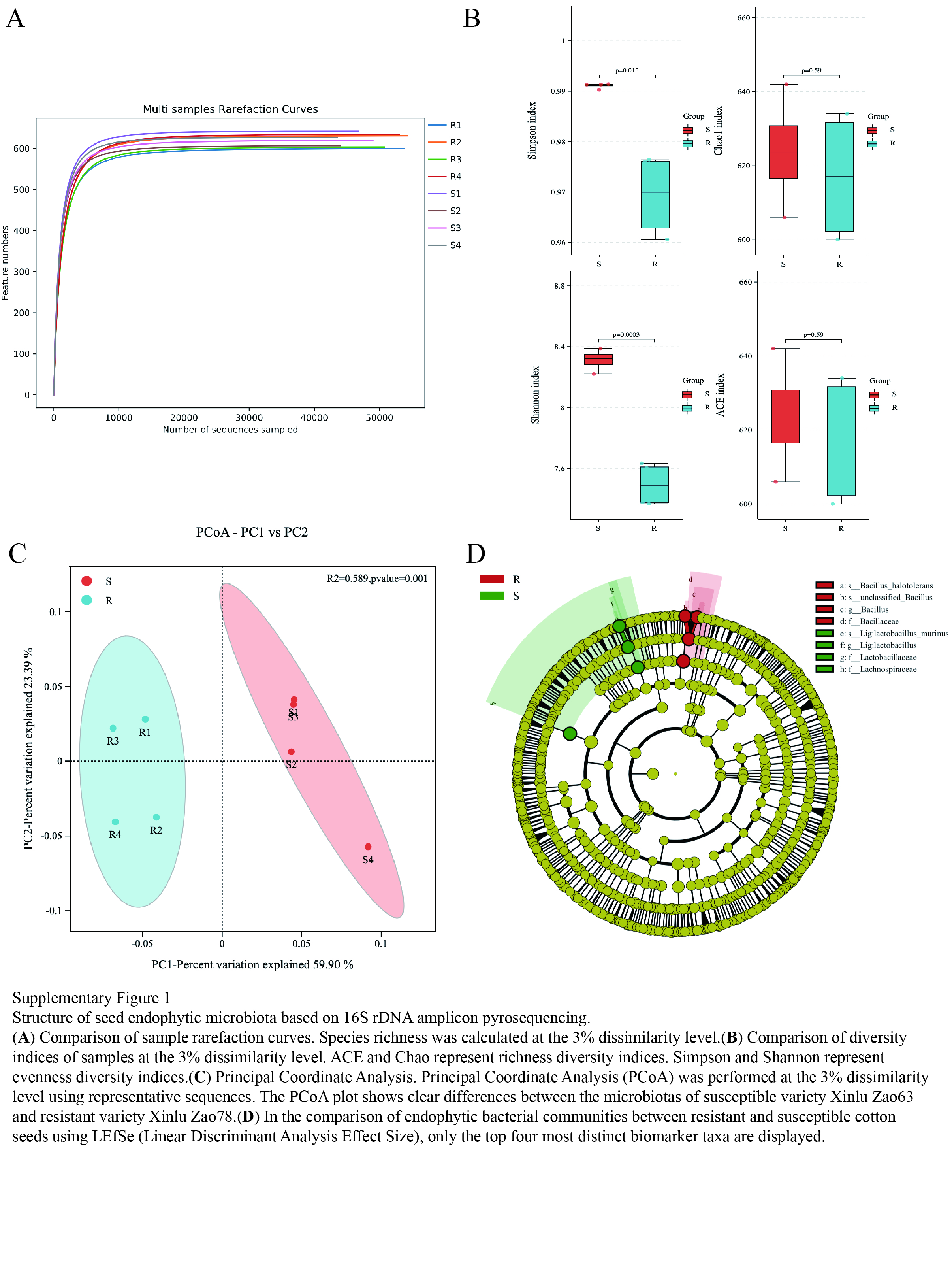

Supplement: Supplementary file 1 — Additional file 1: Supplementary Fig. 1. Structure of seed endophytic microbiota based on 16S rDNA amplicon pyrosequencing. (A) Comparison of sample rarefaction curves. Species richness was calculated at the 3% dissimilarity level.(B) Comparison of diversity indices of samples at the 3% dissimilarity level. ACE and Chao represent richness diversity indices. Simpson and Shannon represent evenness diversity indices.(C) Principal Coordinate Analysis. Principal Coordinate Analysis (PCoA) was performed at the 3% dissimilarity level using representative sequences. The PCoA plot shows clear differences between the microbiotas of susceptible variety Xinlu Zao63 and resistant variety Xinlu Zao78.(D) In the comparison of endophytic bacterial communities between resistant and susceptible cotton seeds using LEfSe (Linear Discriminant Analysis Effect Size), only the top four most distinct biomarker taxa are displayed. [file 12870_2024_4910_MOESM1_ESM.jpg]

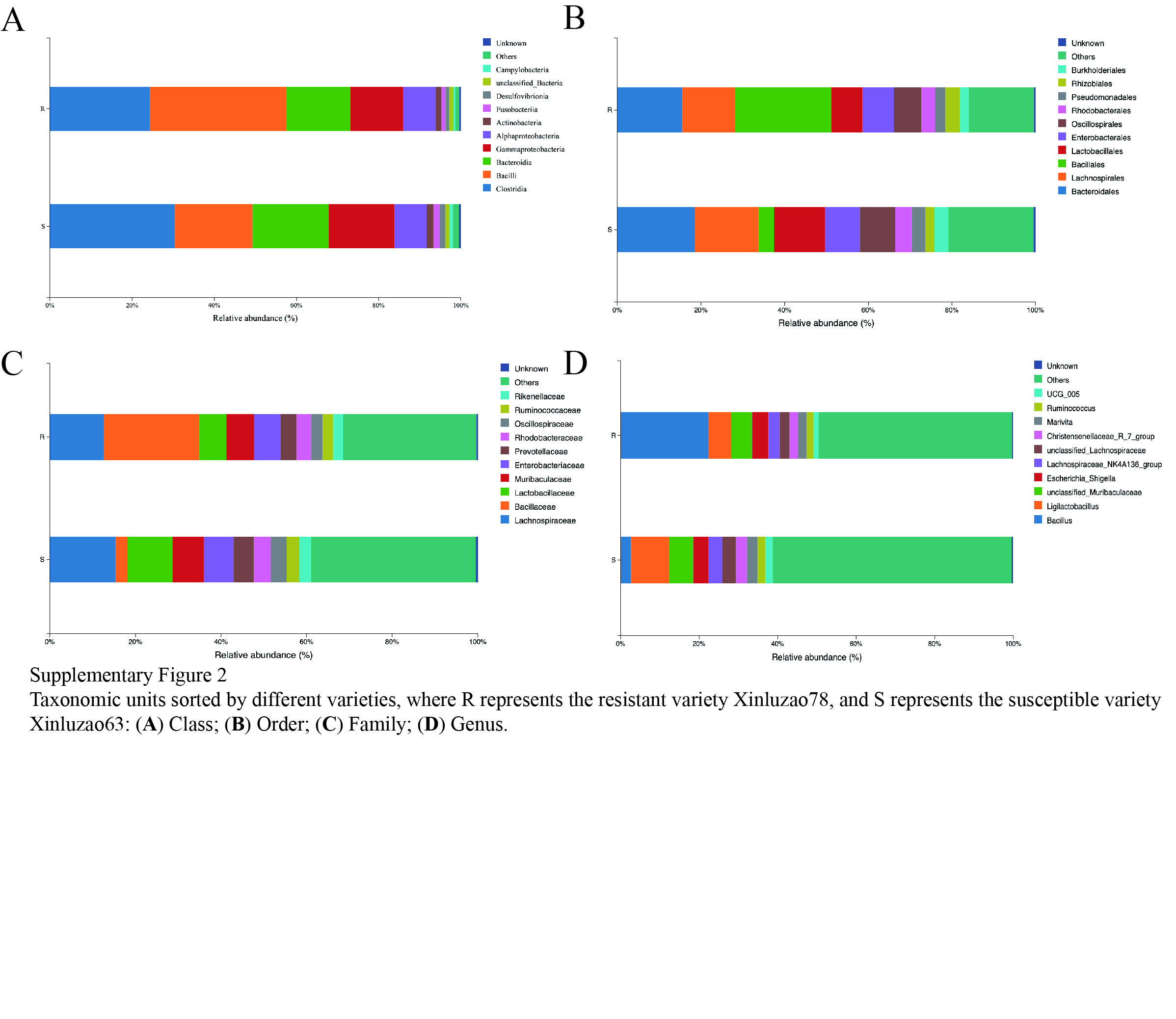

Supplement: Supplementary file 2 — Additional file 2: Supplementary Fig. 2. Taxonomic units sorted by different varieties, where R represents the resistant variety Xinluzao78, and S represents the susceptible variety Xinluzao63: (A) Class; (B) Order; (C) Family; (D) Genus. [file 12870_2024_4910_MOESM2_ESM.jpg]

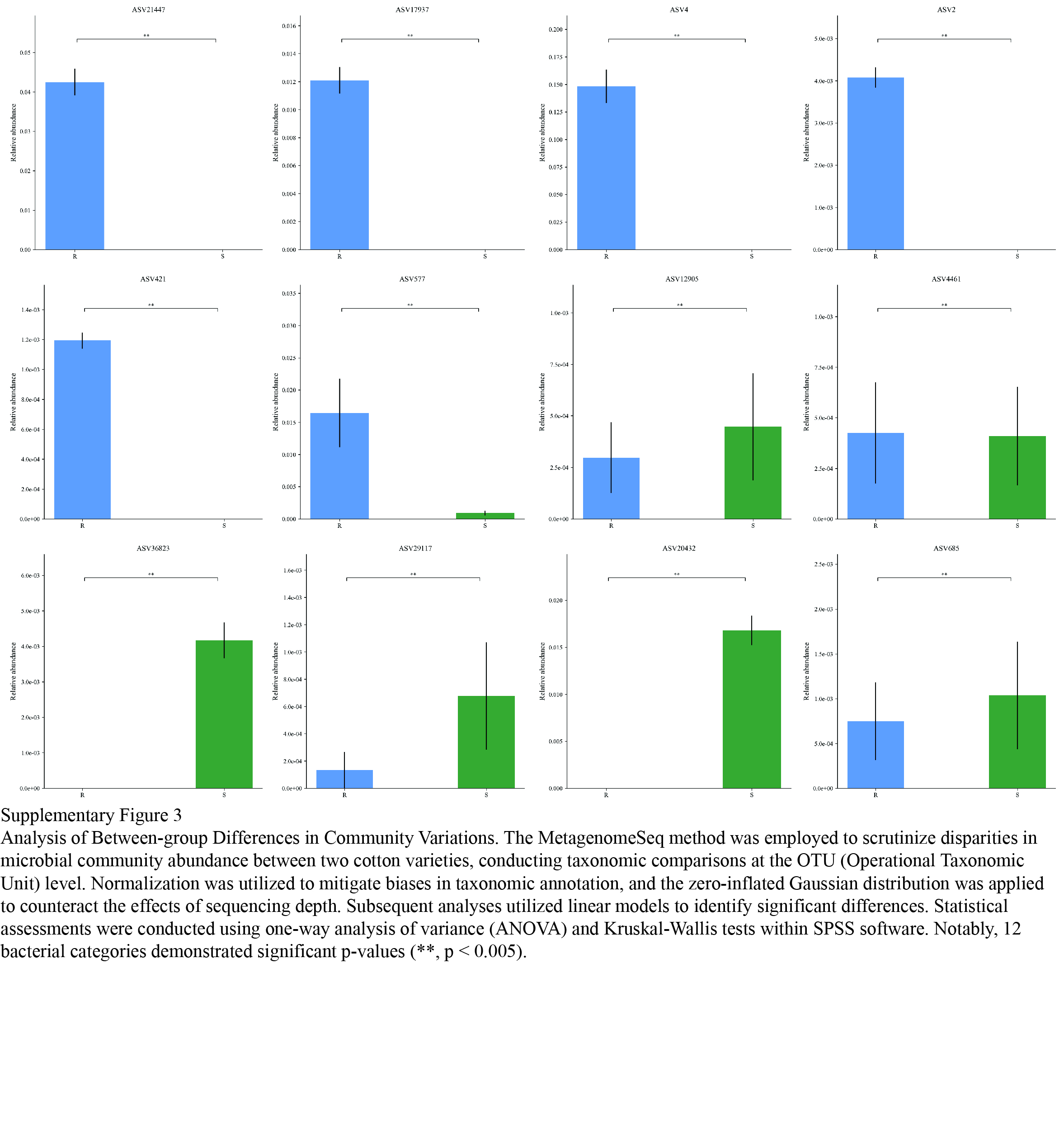

Supplement: Supplementary file 3 — Additional file 3 Supplementary Fig. 3. Analysis of Between-group Differences in Community Variations. The MetagenomeSeq method was employed to scrutinize disparities in microbial community abundance between two cotton varieties, conducting taxonomic comparisons at the OTU (Operational Taxonomic Unit) level. Normalization was utilized to mitigate biases in taxonomic annotation, and the zero-inflated Gaussian distribution was applied to counteract the effects of sequencing depth. Subsequent analyses utilized linear models to identify significant differences. Statistical assessments were conducted using one-way analysis of variance (ANOVA) and Kruskal–Wallis tests within SPSS software. Notably, 12 bacterial categories demonstrated significant p-values (**, p < 0.005). [file 12870_2024_4910_MOESM3_ESM.jpg]

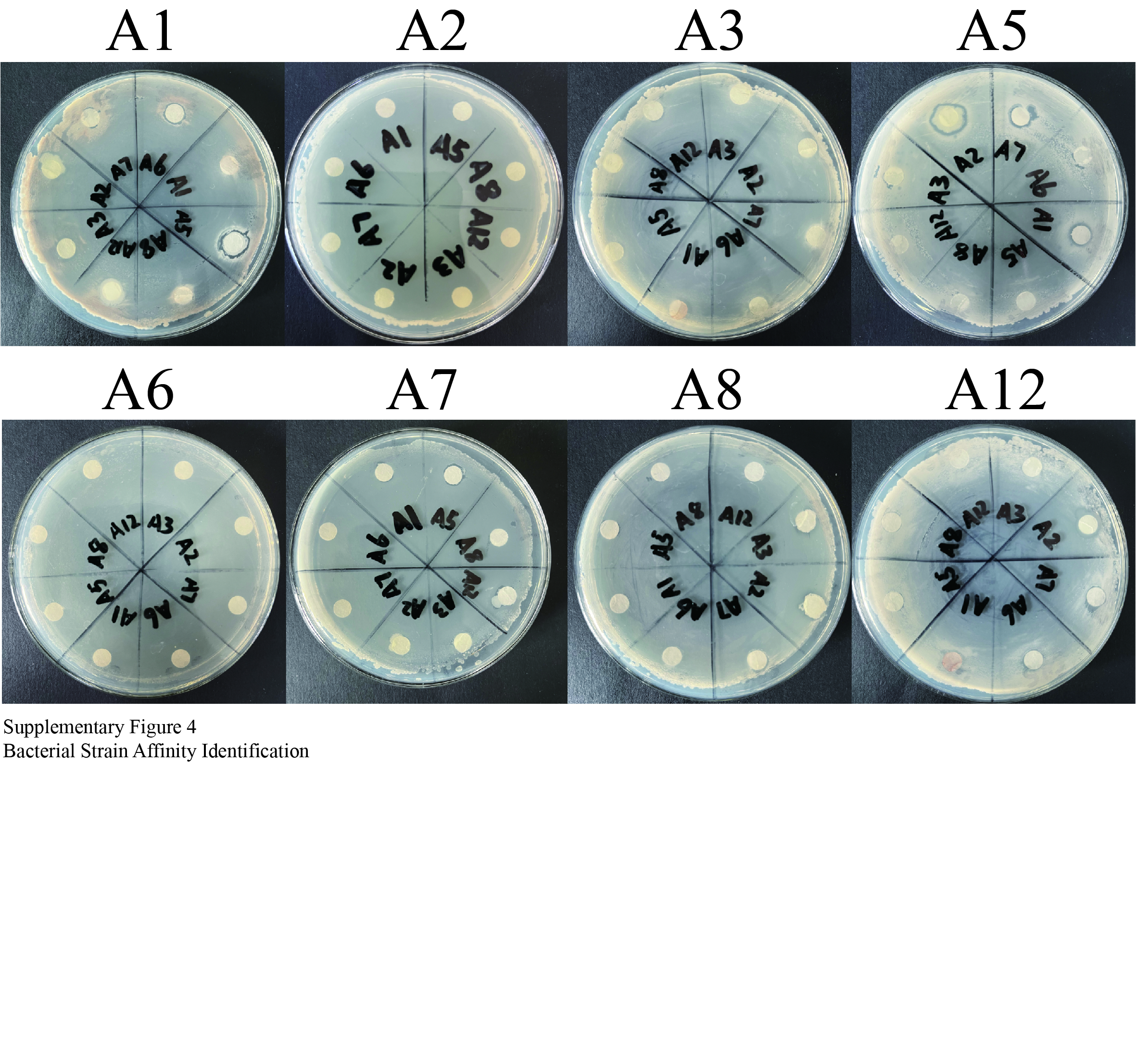

Supplement: Supplementary file 4 — Additional file 4: Supplementary Fig. 4. Bacterial Strain Affinity Identification. [file 12870_2024_4910_MOESM4_ESM.jpg]

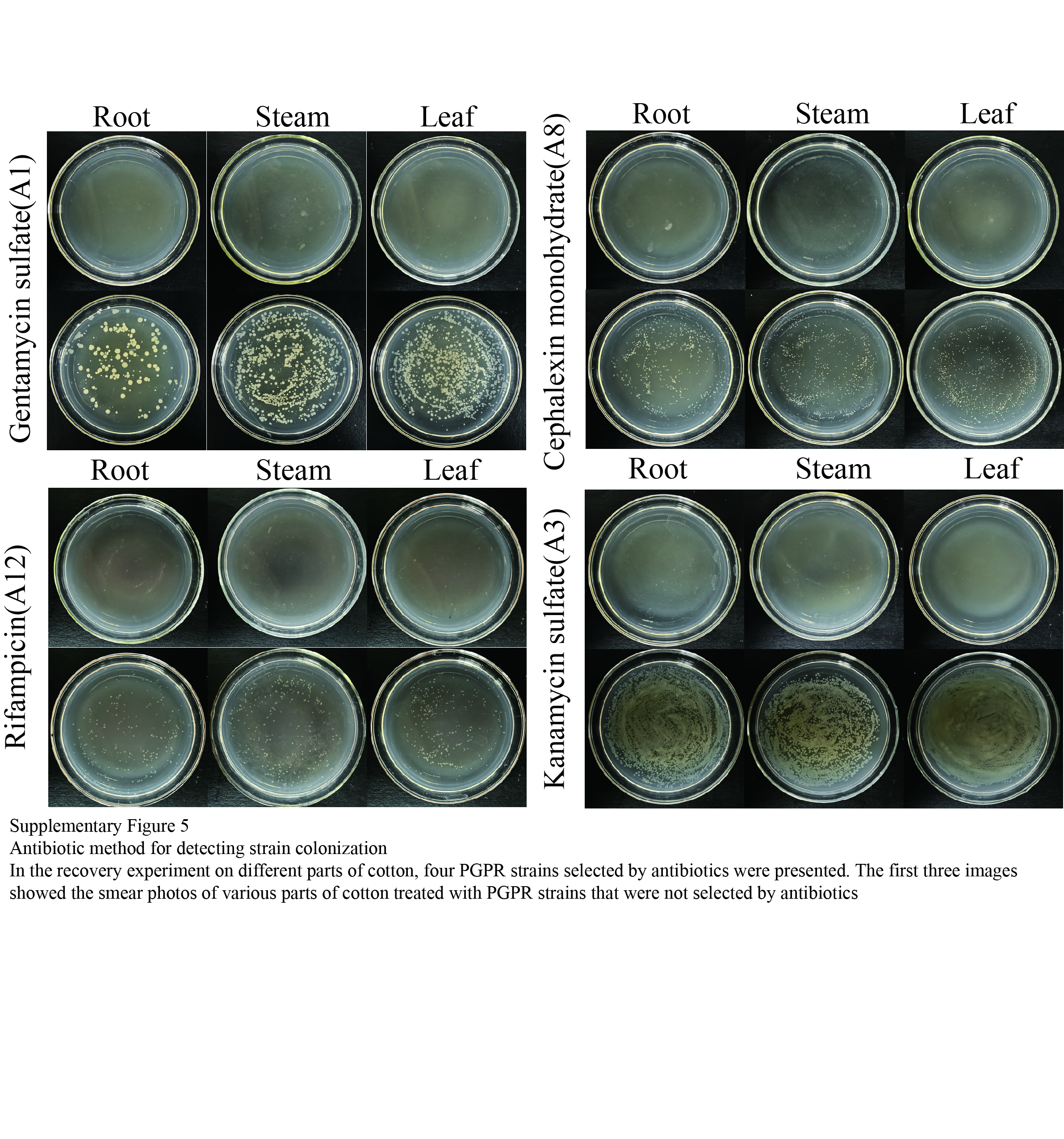

Supplement: Supplementary file 5 — Additional file 5: Supplementary Fig. 5. Antibiotic method for detecting strain colonization. In the recovery experiment on different parts of cotton, four PGPR strains selected by antibiotics were presented. The first three images showed the smear photos of various parts of cotton treated with PGPR strains that were not selected by antibiotics. [file 12870_2024_4910_MOESM5_ESM.jpg]
